# Health Education Modalities and Influencing Factors in Rural Philippine Communities: A Mixed-Methods Study

**DOI:** 10.3390/healthcare14020210

**Published:** 2026-01-14

**Authors:** Andrew Thomas Reyes, Carol Manilay-Robles, Reimund Serafica, Marysol C. Cacciata, Jennifer Kawi, Lorraine S. Evangelista

**Affiliations:** 1 School of Nursing, University of Nevada, Las Vegas, NV 89154, USA; 2 Arleigh Burke Pavilion, McLean, VA 22101, USA; 3 Veterans Affairs Long Beach Healthcare System, Long Beach, CA 90822, USA; 4 Cizik School of Nursing at UTHealth, Houston, TX 77030, USA; jennifer.kawi@uth.tmc.edu; 5 Sue & Bill Gross School of Nursing, University of California, Irvine, CA 92697, USA

**Keywords:** health education, rural communities, community-based health communication, trust, language concordance, Philippines, mixed-methods study

## Abstract

**Background:** Health education is a vital component of preventative care; however, rural Filipino adults often face structural, linguistic, and access barriers to obtaining reliable health information. Designing equitable and culturally relevant health education programs requires understanding which sources are most significant and how context affects them. **Objective:** To identify preferred sources of health education among adults in rural Philippine communities and investigate the contextual factors that influence these preferences. **Methods:** A cross-sectional mixed-methods study included 1203 adults from disadvantaged Luzon and Visayas barangays. Participants completed a self-administered survey on the importance of neighborhood health fairs, native-language instructional tools, and social media. Descriptive statistics (mean ± standard deviation) were used to aggregate importance ratings, and exploratory comparisons were made using paired and independent-samples *t*-tests. A subsample of 60 semi-structured interviews was analyzed using thematic analysis to interpret qualitative data. **Results:** Community health fairs were identified as the primary source of health education, with a mean rating of 8.5 ± 1.6, followed by native-language educational materials, which received a mean rating of 5.5 ± 2.4. In contrast, social media was rated the lowest, with a mean of 3.5 ± 2.3. Preference patterns were consistent across regions and sociodemographic groups, with only slight variation in rating magnitudes. Qualitative analysis revealed four themes influencing source preferences: accessibility and proximity, cultural and linguistic relevance, confidence in local health providers, and structural obstacles to digital access. **Conclusions:** In rural Philippine communities, intimacy, confidence, and cultural congruence influence health education preferences more than online platforms do. Strengthening community-based, locally integrated health education strategies may enhance the reach and contextual relevance of preventive health communication in underserved settings.

## 1. Introduction

Health education plays a vital role in preventive care by enabling individuals to access, understand, assess, and use health information effectively. Access to health information helps individuals to make informed choices and engage in health-promoting behaviors [1]. In many low- and middle-income countries (LMICs), structural barriers hinder health education, including limited access to healthcare providers, remote locations, transportation costs, and a lack of culturally and linguistically appropriate information. These challenges worsen disparities in health literacy and the utilization of preventive services, especially among adults in rural areas where health systems are often decentralized and underfunded.

In the Philippines, health education is typically delivered through local systems like barangay programs and primary care centers. Barangays are the smallest administrative units, similar to villages or neighborhoods. In decentralized settings, community health workers—especially barangay health workers—along with local health institutions, are likely to deliver the most crucial health information and guidance [2]. Recent research in the Philippines indicates that individuals in medically underserved communities rely on local, trusted sources when seeking health information [3]. Complementary qualitative evidence suggests that barangay health workers often serve as the initial point of contact and a link between community members and the larger health system, despite their roles and working conditions being constrained by structural and organizational constraints [4,5]. Evidence from low- and middle-income countries indicates that community health workers promote equity by enhancing access to information and services in ways that are locally credible and practically feasible [6]. Nevertheless, workload and systemic constraints can impact both the implementation process and its long-term sustainability [7].

Meanwhile, digital platforms are increasingly promoted as scalable methods for health education and communication. However, rural communities often face a “digital health divide,” in which the benefits of technology-based health messaging are hindered by poor connectivity, high costs, and limited digital skills [8,9]. In the Philippines, research during the COVID-19 era has also highlighted variability in telemedicine acceptance and intention to use across urban, rural, and remote settings, suggesting that technology adoption is shaped by both access and perceived feasibility within daily life contexts [10]. These realities mean that lower digital channel usage may be due to structural barriers rather than a lack of willingness to engage with technology.

Beyond simply access, trust and the quality of communication are key factors influencing preferences for health information sources. Research on migrant and minority-language groups shows that language barriers can undermine confidence and engagement with health services. In contrast, communicating in a patient’s native language can enhance the experience and motivate participation in care [11,12,13]. Although these insights are largely drawn from settings outside the rural Philippines, they point to a universal principle: providing health information through linguistically and culturally familiar channels enhances trust, practicality, and safety, especially on sensitive issues. In rural Philippine communities, where dialects and cultural norms shape how information is shared, understanding preferences regarding proximity, trust, and language alignment is essential for creating culturally tailored health education strategies.

Despite the increasing focus on health communication and digital health, there is a paucity of mixed-methods evidence detailing (1) the health education sources that adults in rural Philippine communities regard as most significant and trustworthy, and (2) the contextual factors influencing these preferences across various settings. Current research frequently describes health information sources but lacks a consistent analysis of mechanisms—such as proximity, cultural relevance, trust in formal systems, and technological barriers—that may help elucidate preferences for specific modalities. Recognizing this gap is critical for establishing health education programs that improve equal access to credible information and promote preventive health behaviors in underserved barangays.

This study aimed to (1) investigate preferred sources of health education among adults in underserved rural barangays in Luzon and Visayas; and (2) assess contextual factors influencing these preferences, such as accessibility and proximity, cultural and linguistic relevance, trust in formal healthcare, and structural impediments to technology utilization.

## 2. Methods

### 2.1. Study Design and Setting

This study employed a cross-sectional research design in rural Filipino communities located in the Luzon and Visayas regions, conducted from January to June 2017. This study was conducted as part of a broader assessment of health needs [14]. We selected barangays representative of marginalized areas, characterized by a scarcity of clinics, insufficient transportation, and a varied socioeconomic landscape. This was conducted to demonstrate the diversity of health information environments on various islands [15]. Trained research assistants stationed at local barangay health clinics invited all adults aged eighteen and older to participate, explaining the study’s purpose and procedures. Although the survey was offered in both Tagalog and English, every participant chose to complete the English version, likely reflecting their comfort with the language or the predominance of bilingual staff at the clinic [3].

We purposively selected sixty individuals to participate in the survey and subsequently conducted comprehensive, semi-structured interviews to gain insight into the factors that render various media interesting. This subsample was selected because of the diversity in age, gender, and frequency of clinic visits among its participants. Bilingual interviewers conducted interviews at participants’ residences or at nearby, accessible locations. This measure was enacted to safeguard privacy and alleviate fatigue. We evaluated our interview guide with 20 participants and updated it based on their feedback. The focus was on prompting narrators to explore situations in which they sought health information, differentiate between formal and informal sources, and recognize personal and systemic obstacles to accessing trustworthy knowledge. Before analysis, detailed transcripts of the audio recordings were prepared, and participants’ identities were anonymized.

### 2.2. Data Collection

The quantitative component of this study enrolled 1203 adults from purposively selected barangays in Luzon and Visayas. The survey began by collecting demographic data, including age, gender, education, and occupation. Participants were asked to evaluate three primary platforms for health education (community health fairs, native-language print and video materials, and social media content) using a 10-point Likert-type scale to assess the importance of each modality. Before implementation, cognitive pretesting with 40 community members resulted in minor language adjustments to enhance clarity and cultural appropriateness.

The qualitative part included semi-structured interviews with a purposive sample of 60 participants, chosen to maximize variation in age, gender, and clinic visit frequency. An initial pilot of the interview guide with 20 individuals informed iterative improvements. Interviews invited individuals to describe specific occasions when they sought health information, to compare formal versus informal channels, and to discuss both personal and systemic facilitators and barriers to accessing reliable health information. Audio recordings were transcribed verbatim, de-identified, and prepared for analysis.

### 2.3. Data Analysis

Quantitative data were entered into SPSS Statistics version 27 [16] and analyzed using descriptive and inferential statistics. Descriptive statistics, including frequencies, percentages, means, and standard deviations, were used to summarize participant characteristics and ratings of importance for each health education modality. Paired-sample *t*-tests were conducted to compare mean importance ratings across the three focal modalities, and independent-samples *t*-tests were used to examine regional differences between participants residing in Luzon and Visayas.

Additional exploratory independent-samples *t*-tests were performed to assess whether importance ratings varied across selected sociodemographic characteristics, including gender, age category, educational attainment, marital status, employment status, regular physician use, and engagement in community health activities. These subgroup analyses were conducted to evaluate the robustness of overall preference patterns rather than to test a priori hypotheses.

The assumptions of parametric testing were considered appropriate given the large sample size and the approximate normality of the rating distributions. Statistical significance was evaluated using a two-sided alpha threshold of *p* < 0.05. Effect sizes were calculated using Cohen’s *d* and interpreted using conventional benchmarks (0.2 = small, 0.5 = medium, 0.8 = large). Subgroup analyses were exploratory and were not adjusted for multiple comparisons.

Qualitative interview transcripts were analyzed via reflexive thematic analysis. The audio-recorded interviews were transcribed word-for-word, anonymized, and then imported into NVivo (Version 10) for data organization and coding. Two members of the research team independently examined an initial batch of transcripts to familiarize themselves with the data and develop preliminary codes using an inductive method [17].

The initial codebook was created through open coding and iterative discussions, with codes grouped into broader categories to identify common patterns. Two coders independently used the refined codebook on a second set of transcripts, meeting regularly to compare results, resolve differences, and refine definitions. Interrater reliability exceeded 85%, indicating high consistency. Once agreement was reached, the final codebook was applied to the entire dataset. Codes were then clustered into themes that captured shared meanings related to health information-seeking behaviors, perceived trustworthiness of sources, cultural and linguistic relevance, and structural barriers to access. Theme development was an iterative process that involved repeated comparisons of transcripts to ensure coherence, internal consistency, and clear differentiation among themes. To improve analytical rigor, contrasting perspectives were identified, and themes were refined to include both prominent and opposing experiences [18]. Quotations were added to illustrate each theme, increasing transparency and credibility. The research team included investigators with prior experience conducting community-based health research in Filipino populations, which informed reflexive discussions throughout the analytic process regarding interpretation, language context, and researcher positionality.

### 2.4. Ethical Considerations

This study was approved by the Institutional Review Boards of the University of California, Irvine (Protocol #2024-123, 17 March 2018) and the University of the Philippines Manila (Protocol #UPM-IRB-2024-45, 4 January 2017). Informed consent was obtained before all data collection. To safeguard privacy, survey data and transcripts were anonymized and stored on encrypted servers accessible only to the core study team. This tight ethical framework ensured that our study of health and education preferences adhered to the highest scientific standards while also respecting the participants’ rights.

## 3. Results

### 3.1. Sample Characteristics

The survey was completed by 1203 individuals from rural barangays in the Visayas and Luzon regions. The participants, who ranged in age from 18 to over 80, had diverse educational backgrounds, including those who had not completed primary school and those who had earned advanced degrees. Most individuals supported themselves through informal work, small-scale commerce, or farming, and the population was nearly equally divided by gender. Many respondents reported visiting their local barangay health clinic at least once in the past year. This implies that these clinics continue to serve as the primary method of healthcare access for individuals. Stratified descriptive and inferential analyses by key sociodemographic characteristics are presented in the Supplementary Materials (Tables S1–S6).

### 3.2. Quantitative Findings

Table 1 summarizes mean importance ratings for community health fairs, native-language educational materials, and social media among all 1203 participants, along with regional (Luzon vs. Visayas) and selected sociodemographic stratifications. Community health fairs were consistently rated as the most important source of health education (M = 8.5, 95% CI [8.41, 8.59]), with nearly identical ratings in Luzon (M = 8.6) and Visayas (M = 8.4) and no statistically significant regional difference (Δ = 0.2, *p* = 0.12). Native-language print and video materials received moderate importance ratings overall (M = 5.5, 95% CI [5.43, 5.57]), with a small but statistically significant preference among participants in Luzon compared with Visayas (Δ = 0.4, *p* = 0.04). Social media was rated lowest across regions (M = 3.5, 95% CI [3.41, 3.59]). Paired comparisons confirmed that community health fairs were rated significantly higher than both native-language materials and social media (*p* < 0.001), and that native-language materials were rated significantly higher than social media (*p* < 0.001).

Exploratory stratified analyses were conducted to examine whether preferences varied across sociodemographic characteristics, including gender, age category, educational attainment, marital status, employment status, regular physician use, and engagement in community health activities (Table 1 and Tables S1–S6). Across all subgroups, the relative ranking of modalities remained stable, with community health fairs consistently rated highest. Although several subgroup comparisons reached statistical significance—particularly by gender, educational attainment, and regular physician use—the magnitude of these differences was small (Cohen’s *d* generally < 0.25) and did not meaningfully alter the overall preference pattern. Age-based differences were minimal and largely non-significant (Table S2). Full stratified results are provided in the Supplementary Materials (Tables S1–S6).

### 3.3. Qualitative Findings

In the in-depth interviews, four interrelated themes emerged to explain these patterns (see Table 2). First, accessibility and proximity drove many choices: having a community health worker “just down the road” removed travel barriers and built trust. Second, cultural resonance mattered—participants favored sources that spoke their dialect and understood local customs. Third, several individuals choose not to utilize formal care; some sought assistance from neighbors, family, or religious organizations due to financial concerns or negative past experiences with formal care. Ultimately, technical issues such as inadequate internet connectivity and limited digital literacy hindered the majority from accessing mobile-based assistance, despite their genuine need for reminders or advice via phone. These themes underscore the ongoing significance of specific health education channels in rural regions of the Philippines and highlight opportunities to engage a broader audience.

## 4. Discussion

This mixed-methods study examined preferences for health education modalities among individuals living in underprivileged barangays in Luzon and Visayas. Participants uniformly favored in-person, community-oriented sources—especially community health fairs and nearby clinics—over native-language resources and internet platforms, regardless of geography or sociodemographic group. Qualitative findings elucidated the mechanisms driving these preferences, emphasizing proximity, cultural and language affinity, trust in local health practitioners, and structural barriers to digital access. The findings indicate that health education preferences in rural Philippine communities are predominantly influenced by congruence with daily social and structural realities rather than by the use of innovative communication technology.

### 4.1. Comparison with Prior Evidence from LMIC and Immigrant Community Settings

Our findings align with a growing body of evidence from LMICs demonstrating that community-embedded health communication channels remain central in settings characterized by geographic dispersion, limited infrastructure, and constrained access to formal healthcare.

Research from LMICs consistently indicates that community health workers and local clinics act as trusted intermediaries because they blend health expertise with social closeness, cultural familiarity, and ongoing presence [6,19]. These factors emphasize participants’ focus on convenience and trust in others as vital when seeking and using health information.

Studies on immigrant and migrant health show that clear communication—via language matching and cultural insight—is crucial for establishing trust and motivating involvement with health data. When communication is not in the preferred language, it can reduce understanding, undermine confidence, and delay the use of preventive services, even in wealthier settings [11,20]. These dynamics align with our study’s qualitative results, in which participants valued health education from individuals who spoke their dialect, understood local customs, and were part of community networks.

The limited support for digital platforms noted here aligns with existing research in LMICs, which highlights ongoing structural challenges, including poor connectivity, high costs, low digital literacy, and mistrust of technology-based care [9]. Participants tend to make rational decisions by avoiding technology when in-person options are more reliable and accessible. Understanding this is vital for planning health systems, as ignoring infrastructure upgrades and trust-building efforts could worsen health inequalities if digital solutions are implemented without tackling these core issues.

### 4.2. Integration of Quantitative and Qualitative Findings

The integration of quantitative evaluations and qualitative narratives elucidates both the favored health education strategies and the fundamental reasons for their sustained application across diverse sociodemographic groups. Exploratory subgroup analysis revealed statistically significant differences; however, the effect sizes were small and did not significantly affect the overall ranking of modalities. Qualitative findings contextualize these patterns by illustrating that trust, proximity, and cultural compatibility serve as overarching mechanisms that extend beyond individual attributes such as age or education. This convergence highlights the value of mixed-methods approaches in distinguishing statistically detectable variation from substantively meaningful differences in lived experience.

### 4.3. Implications for Health Education Practice and Systems

From the perspective of health systems and public health, these findings indicate that effective health education strategies in rural Philippine settings should focus on strengthening existing community-based infrastructure rather than relying primarily on digital dissemination or passive distribution of materials. Community health fairs and barangay-based activities serve as both information dissemination venues and relational environments that facilitate the establishment and reinforcement of trust. Materials in the native language can improve understanding and retention when integrated into these contexts. Conversely, digital initiatives are typically executed in phases, involving investments in infrastructure, digital literacy, and community engagement.

### 4.4. Strengths and Limitations

This study is enhanced by a substantial, geographically diverse sample and the amalgamation of quantitative and qualitative data, offering a comprehensive overview of health education preferences. The utilization of independent coding and iterative theme development improved the reliability of qualitative findings, while exploratory stratified analyses demonstrated the strength of preference patterns across socioeconomic groups.

Several limitations must be considered when analyzing these findings. Initially, the cross-sectional design and dependence on self-reported data introduce potential biases related to recall accuracy and social desirability. Secondly, the use of purposive and convenience sampling techniques restricts the extent to which findings can be generalized beyond the rural barangays included in this research.

The lack of specific metrics for health literacy, digital access, and technology use limited our ability to accurately assess the impact of these factors on preferences for health education modalities. Moreover, the use of traditional or alternative medicine was not thoroughly evaluated. The prevalence of informal, culturally ingrained health practices in many rural communities leads to a limited understanding of the relationship between these practices and informal health information channels, which, in turn, influences the choice of reliable sources.

Ultimately, while qualitative findings emphasized the significance of communication in indigenous languages, the surveys were conducted in English, which may have affected participation, understanding, or response tendencies. Exploratory subgroup analyses were performed to assess the consistency of findings across sociodemographic categories; however, these analyses did not adjust for multiple comparisons and should be interpreted with caution. The identified limitations highlight the importance of future research employing longitudinal designs, language-appropriate instruments, and comprehensive assessments of digital access and informal caregiving practices.

Despite these challenges, the study makes a significant contribution to the growing body of research on health education in rural areas of LMICs [21]. It is clear from the findings that devising communication strategies is essential, not only to be fair but also to be culturally sensitive and flexible enough to accommodate the particular conditions in each community [22]. To accomplish this, they focus on the community’s goals and investigate the factors that influence decisions related to health matters [23].

### 4.5. Future Directions

Future research must assess hybrid health education models that combine community-based engagement with low-tech or supported digital tools. Addressing infrastructural and access barriers may be necessary for digital health tools to be equitably implemented in rural communities [24]. Longitudinal or experimental designs should be employed to evaluate their effects on health literacy and preventive behaviors [25]. Extending research to more regions and linguistic groups will enhance the understanding of the generalizability of these findings and guide the development of scalable, equity-focused health education strategies.

## 5. Conclusions

Adults residing in underserved barangays in Luzon and Visayas consistently favored in-person, community-based sources of health education over native-language materials and digital platforms. Preferences were influenced by proximity, trust in local health actors, cultural and linguistic alignment, and structural barriers to digital access, remaining consistent across sociodemographic groups. The findings highlight the significance of community-centered health education strategies that leverage established local infrastructure and tackle ongoing inequities in access to formal care and digital resources.

## Figures and Tables

**Table 1:** Stratified differences in importance ratings for health education modalities by region and sociodemographic characteristics (N = 1203).

Stratification Variable	Comparison Groups	Direction of Difference	Statistical Significance	Effect Size (Cohen’s *d*)	Summary Interpretation
Region	Luzon vs. Visayas	Luzon is slightly higher for native-language materials; similar for fairs	*p* = 0.04	Small (≈0.10–0.15)	Regional differences minimal
Gender	Female vs. Male	Females rated fairs and native-language programs higher	*p* < 0.01	Small (≈0.17–0.22)	Modest gender variation
Age	<40 vs. ≥40 years	No consistent differences	ns	Trivial (≤0.10)	Preferences are stable across age
Education	≥High school vs. <High school	Higher education rated all modalities higher	*p* < 0.05	Small (≈0.15–0.25)	Engagement effect
Marital status	Married/partnered vs. single/divorced/widowed	Married participants rated fairs higher	*p* < 0.01	Small (≈0.15–0.18)	Limited practical impact
Employment	Employed vs. unemployed	Employed rated materials slightly higher	*p* = 0.001	Small (≈0.19)	Minor socioeconomic gradient
Regular physician use	Yes vs. No	Regular users rated all modalities higher	*p* < 0.01	Small (≈0.12–0.21)	Access-related engagement
Community health engagement	Yes vs. No	Engaged participants rated fairs higher	*p* < 0.01	Small (≈0.18)	Expected pattern

Notes: Table 1 summarizes exploratory subgroup comparisons of importance ratings for health education modalities. The direction of difference indicates the group with higher mean ratings. Statistical significance reflects independent-samples *t*-tests (two-sided). *p* < 0.05 was considered statistically significant; ns = not significant. Effect sizes are reported as approximate ranges of Cohen’s *d* derived from subgroup analyses and are interpreted as small (0.2), medium (0.5), or large (0.8). Subgroup analyses were exploratory and not adjusted for multiple comparisons. Full stratified results are provided in Supplementary Tables S1–S6. Reported effect size ranges reflect variation across modalities within each subgroup rather than a single pooled estimate.

**Table 2:** Primary qualitative themes shaping source preference with example quotes.

Theme	Description	Example Quotes
Accessibility and proximity	Participants favored channels located in their barangay, as face-to-face contact lowered travel time and costs, and fostered trust.	“The health worker lives just down the road so that I can ask questions any day.” “I don’t have to take a jeepney to town; the clinic here is easy to reach.”
Cultural resonance	Health messages delivered by someone “from our community” felt more relatable and actionable.	“When she speaks in our dialect, I understand better what I need to do.” “He knows our traditions, so his advice fits how we live.”
Reluctance toward formal care	Financial constraints and past negative experiences led many to rely first on informal networks (family, neighbors) before seeking clinics.	“My cousin taught me to use herbal tea before I ever went to the clinic.” “Hospitals cost too much, so I ask my neighbor, who’s a nurse, for tips.”
Technological barriers	Inconsistent internet access and limited digital literacy prevented most digital tools from being regularly used, despite interest in mobile messaging.	“My phone can’t load the health app; the signal always drops.” “I don’t know how to use those online videos; I just stick with radio news.”

Note: Themes were derived using thematic analysis as described in Section 2.3. Quotations are de-identified and may be lightly edited for clarity and brevity; ellipses indicate omitted text.

## Data Availability

To safeguard privacy, survey data and transcripts were anonymized and stored on encrypted servers accessible only to the core study team.

## Supplementary Materials

The following supporting information can be downloaded at: https://www.mdpi.com/article/10.3390/healthcare14020210/s1, Table S1: Importance ratings of health education modalities by gender; Table S2: Importance ratings by age category; Table S3: Importance ratings by educational attainment; Table S4: Importance ratings by marital status; Table S5: Importance ratings by regular physician use; Table S6: Importance ratings by engagement in community health activities.
