# Supplementary material for: Health Education Modalities and Influencing Factors in Rural Philippine Communities: A Mixed-Methods Study
Source: Healthcare (Basel). 2026 Jan 14;14(2):210. doi: 10.3390/healthcare14020210 (PMC12841488; doi:10.3390/healthcare14020210)
Supplement: Supplementary file 1 [file healthcare-14-00210-s001.zip › healthcare-4064156-supplementary.pdf]

Supplementary Tables

This Supplementary Materials file provides detailed results from exploratory stratified analyses aimed at assessing the robustness of health education modality preferences across key sociodemographic characteristics. These analyses complement the main findings presented in Table 1 and Section 3.2 of the manuscript and are intended to provide additional transparency without expanding the primary Results section. The supplementary tables include independent-samples comparisons of importance ratings for community health fairs, native-language educational materials, and native-language health education programs across gender, age category, educational attainment, marital status, employment status, regular physician use, and engagement in community health activities. All subgroup analyses are exploratory and were not adjusted for multiple comparisons.

Table S1. Importance Ratings of Health Education Modalities by Gender (N = 1,202)

Modality	Male (n = 426) Mean ± SD	Female (n = 776) Mean ± SD	Mean Difference	*p*-value	Cohen's *d*
Community health fairs	6.64 ± 2.05	7.09 ± 2.01	−0.45	<.001	0.22
Native-language materials	6.38 ± 2.87	6.60 ± 2.11	−0.22	.13	0.09
Native-language programs	6.46 ± 2.18	7.10 ± 4.43	−0.65	.005	0.17

Note: Importance ratings were assessed using a 10-point Likert-type scale. Independent-samples *t*-tests were conducted for exploratory subgroup comparisons. Effect sizes are reported using Cohen's *d* and interpreted as small (0.2), medium (0.5), or large (0.8). Analyses were not adjusted for multiple comparisons. Differences did not alter the overall ranking of preferred health education modalities.

Table S2. Importance Ratings of Health Education Modalities by Age Category (N = 1,202)

Modality	<40 years (n = 380) Mean ± SD	≥40 years (n = 822) Mean ± SD	Mean Difference	*p*-value	Cohen's *d*
Community health fairs	6.97 ± 2.27	6.91 ± 1.92	0.06	.65	0.03
Native-language materials	6.50 ± 2.37	6.53 ± 2.42	−0.03	.82	0.01
Native-language programs	7.18 ± 5.27	6.73 ± 2.87	0.45	.06	0.12

Note: Importance ratings were assessed using a 10-point Likert-type scale. Independent-samples *t*-tests were conducted for exploratory subgroup comparisons. Effect sizes are reported using Cohen's *d* and interpreted as small (0.2), medium (0.5), or large (0.8). Analyses were not adjusted for multiple comparisons. Differences did not alter the overall ranking of preferred health education modalities.

Table S3. Importance Ratings of Health Education Modalities by Educational Attainment (N = 1,202)

Modality	<High School (n = 273) Mean ± SD	≥High School (n = 929) Mean ± SD	Mean Difference	*p*-value	Cohen's *d*
Community health fairs	6.66 ± 1.72	7.01 ± 2.11	−0.35	.01	0.17
Native-language materials	6.06 ± 1.82	6.66 ± 2.54	−0.60	<.001	0.25
Native-language programs	6.42 ± 4.02	7.01 ± 3.72	−0.58	.03	0.15

Note: Importance ratings were assessed using a 10-point Likert-type scale. Independent-samples *t*-tests were conducted for exploratory subgroup comparisons. Effect sizes are reported using Cohen's *d* and interpreted as small (0.2), medium (0.5), or large (0.8). Analyses were not adjusted for multiple comparisons. Differences did not alter the overall ranking of preferred health education modalities.

Table S4. Importance Ratings of Health Education Modalities by Marital Status (N = 1,202)

Modality	Married/Partnered (n = 704) Mean ± SD	Single/Divorced/ Widowed (n = 498) Mean ± SD	Mean Difference	*p*-value	Cohen's *d*
Community health fairs	7.07 ± 1.96	6.74 ± 2.12	0.33	.006	0.16
Native-language materials	6.62 ± 2.13	6.38 ± 2.75	0.24	.09	0.10
Native-language programs	7.04 ± 4.30	6.64 ± 2.94	0.40	.07	0.11

Note: Importance ratings were assessed using a 10-point Likert-type scale. Independent-samples *t*-tests were conducted for exploratory subgroup comparisons. Effect sizes are reported using Cohen's *d* and interpreted as small (0.2), medium (0.5), or large (0.8). Analyses were not adjusted for multiple comparisons. Differences did not alter the overall ranking of preferred health education modalities.

Table S5. Importance Ratings of Health Education Modalities by Regular Clinic Use (N = 1,200)

Modality	No (n = 503) Mean ± SD	Yes (n = 697) Mean ± SD	Mean Difference	*p*-value	Cohen's *d*
Community health fairs	6.68 ± 1.82	7.11 ± 2.17	−0.44	<.001	0.21
Native-language materials	6.28 ± 2.55	6.69 ± 2.29	−0.41	.004	0.17
Native-language programs	6.62 ± 3.79	7.06 ± 3.80	−0.44	.048	0.12

Note: Importance ratings were assessed using a 10-point Likert-type scale. Independent-samples *t*-tests were conducted for exploratory subgroup comparisons. Effect sizes are reported using Cohen's *d* and interpreted as small (0.2), medium (0.5), or large (0.8). Analyses were not adjusted for multiple comparisons. Differences did not alter the overall ranking of preferred health education modalities.

Table S6. Importance Ratings of Health Education Modalities by Community Health Engagement (N = 1,202)

Modality	Not Engaged (n = 978) Mean ± SD	Engaged (n = 224) Mean ± SD	Mean Difference	*p*-value	Cohen's *d*
Community health fairs	6.86 ± 2.12	7.24 ± 1.59	−0.38	.01	0.18
Native-language materials	6.49 ± 2.55	6.68 ± 1.62	−0.20	.27	0.08
Native-language programs	6.92 ± 4.14	6.66 ± 1.57	0.27	.34	0.07

Note: Importance ratings were assessed using a 10-point Likert-type scale. Independent-samples *t*-tests were conducted for exploratory subgroup comparisons. Effect sizes are reported using Cohen's *d* and interpreted as small (0.2), medium (0.5), or large (0.8). Analyses were not adjusted for multiple comparisons. Differences did not alter the overall ranking of preferred health education modalities.
